# Cholesteryl Esters Are Elevated in the Lipid Fraction of Bronchoalveolar Lavage Fluid Collected from Pediatric Cystic Fibrosis Patients

**DOI:** 10.1371/journal.pone.0125326

**Published:** 2015-04-28

**Authors:** Daniel C. Ma, Alexander J. Yoon, Kym F. Faull, Robert Desharnais, Edith T. Zemanick, Edith Porter

**Affiliations:** 1 Department of Biological Sciences, California State University Los Angeles, Los Angeles, California, United States of America; 2 Pasarow Mass Spectrometry Laboratory, David Geffen School of Medicine at the University of California Los Angeles, Los Angeles, California, United States of America; 3 Department of Pediatrics, University of Colorado School of Medicine, Aurora, Colorado, United States of America; Weill Medical College of Cornell University, UNITED STATES

## Abstract

**Background:**

Host-derived lipids including cholesteryl esters (CEs) such as cholesteryl linoleate have emerged as important antibacterial effectors of innate immunity in the airways and cholesteryl linoleate has been found elevated in the context of inflammation. Cystic fibrosis (CF) patients suffer from chronic infection and severe inflammation in the airways. Here, we identified and quantified CEs in bronchoalveolar lavage fluid (BALF) from CF patients and non-CF disease controls, and tested whether CE concentrations are linked to the disease.

**Materials and Methods:**

CEs in BALF from 6 pediatric subjects with CF and 7 pediatric subjects with non-CF chronic lung disease were quantified by mass spectral analysis using liquid chromatography coupled with tandem mass spectrometry and multiple reaction monitoring. BALFs were also examined for total lipid, total protein, albumin, and, as a marker for inflammation, human neutrophil peptide (HNP) 1–3 concentrations. Statistical analysis was conducted after log 10 transformation of the data.

**Results:**

Total lipid/protein ratio was reduced in CF BALF (*p* = 0.018) but the concentrations of CEs, including cholesteryl linoleate, were elevated in the total lipid fraction in CF BALF compared to non-CF disease controls (*p* < 0.050). In addition, the concentrations of CEs and HNP1-3 correlated with one another (p < 0.050).

**Conclusions:**

The data suggests that the lipid composition of BALF is altered in CF with less total lipid relative to protein but with increased CE concentrations in the lipid fraction, likely contributed by inflammation. Future longitudinal studies may reveal the suitability of CEs as a novel biomarker for CF disease activity which may provide new information on the lipid mediated pathophysiology of the disease.

## Introduction

Cystic fibrosis (CF), one of the most common autosomal recessive genetic disorders, is caused by mutations in the cystic fibrosis transmembrane conductance regulator (CFTR) gene coding for a chloride channel that resides on the apical surface of epithelial cells where it regulates ion transport and hydrates the airways [1]. The hallmark of CF is viscous mucus and defective mucociliary clearance in the respiratory tract [2]. Affected individuals suffer chronic airway infections with characteristic pathogens including *Pseudomonas aeruginosa*, as well as chronic neutrophil-driven inflammation [3]. Gradual remodeling of the lung architecture ultimately leads to lung failure and premature death [3]. The pathogenesis of opportunistic infections with *P*. *aeruginosa* is multifactorial and involves a breach of general and innate immune defenses. Some aspects of impaired defenses in the CF airway affect epithelial cells and include defective bacterial clearance [4], defective apoptotic machinery [5], or dysregulated or inactivated antimicrobial (poly)peptides secretion [6–9].

More recently, lipids have emerged as effector molecules of innate immunity with direct antimicrobial activity when applied alone [10] or in synergism with antimicrobial (poly)peptides [11,12]. For example, the linoleate and arachidonate cholesteryl esters (CEs), which are present in human nasal fluid, exert antimicrobial activity against *P*. *aeruginosa*, and the non-polar lipid component of nasal fluid exhibits synergistic activity with the antimicrobial peptide HNP2 [13]. As recently shown, CEs and non-polar lipids are elevated in sinus washes obtained from chronic rhinosinusitis patients [14] whereby potential sources for CEs are epithelial cells [13,14] as well as monocytes and macrophages [15–17].

CF patients require lifelong and complex therapy. Outcomes are variable with some patients remaining relatively stable over many years, while others experience more rapid disease progression in childhood [18]. Lung function is the most commonly used surrogate outcome measure in CF clinical trials and frequently guides clinical practice [19,20]. As treatment of CF improves, new and more sensitive biomarkers of disease activity are needed to detect treatment effects and predict those at risk for more rapid disease progression [21]. Airway neutrophil elastase activity has recently been shown to be associated with subsequent lung function decline [22] and airway structural injury [23]. Other suggested markers include sweat chloride, which appears to be useful in a subset of CF patients treated with CFTR modulators [24], and miRNA’s measured in nasal epithelial tissues, although their clinical significance has yet to be established [25,26]. Considering the role of antimicrobial CEs in innate immunity and inflammation, we sought to test whether CEs were increased in CF BALF compared to non-CF disease control BALF and could also be used as biomarkers of CF.

## Materials and Methods

### Ethics Statement

All human subjects materials used in this study were collected under full institutional review board approval for studies with human subjects (Colorado Multiple IRB# 99–113) and appropriate written consent.

### Human Specimens

Banked BALF from 6 cystic fibrosis (CF) pediatric subjects and 7 non-CF disease control subjects (Non-CF) was used with personal identifiers removed [27]. Non-CF disease control subjects included patients who were undergoing a clinical bronchoscopy for pulmonary indications other than CF. Immediately following collection, BALF samples were centrifuged (250 × *g*, 10 min, 4°C), and the supernatant was transferred to a sterile polypropylene tube and centrifuged again (4000 × *g*, 20 min, 4°C). The remaining supernatant was further clarified by filtration through a 0.2 μm filter. The clarified samples were then stored at -70°C. **Table 1** summarizes the study population and the BALF characteristics.

**Table 1:** Description of Study Population and BALF Characteristics.

Disease		Subject Identifier	Sex	Age (y)	BALF Volume (mL)	Infection Status ^e^	Nucleated Cells/μL	Red Blood Cells/μL	Neutrophils/μL
Non-CF	CLD ^a^/ILD ^b^	1	Female	11.7	10	Negative	548	25	33
	Stenosis ^c^	3	Male	4.7	8	Positive	134	18	1
	NEHI^d^	4	Male	1.8	7	Positive	77	10	0
	ILD	6	Male	10.1	8.5	Negative	321	136	125
	ILD	9	Female	4.8	8	Negative	495	40	45
	ILD	10	Female	9.5	7	Negative	220	242	77
	ILD	12	Female	2.2	7	Negative	251	735	38
CF ^f^	F508/F508	2	Female	16.6	25	Negative	193	18	69
	F508/F508	5	Female	7.6	10	Positive	1495	525	1181
	F508/F508	7	Female	16.4	12	Positive	783	200	532
	F508/G542X	8	Male	13	12	Negative	386	59	274
	F508/F508	11	Male	13.9	35	Negative	2725	2450	1281
	F508/F508	13	Female	12.1	23	Positive	4808	4733	4183

^a^ Chronic lung disease;

^b^ Interstitial lung disease;

^c^ Subglottic;

^d^ Neuroendocrine hyperplasia of infancy;

^e^ Negative indicates no growth or normal microbiota isolated, positive indicates isolation of probable pathogens;

^f^ CF genotype is given.

### Cholesteryl Ester Standards

Cholesteryl arachidonate, linoleate, oleate, palmitate, stearate, and octanoate-1- ^13^C_1_ were purchased from Sigma-Aldrich (St. Louis, MO), and stock solutions were prepared in dichloromethane at 1 mg/mL and stored under an atmosphere of N_2_ at -20°C (**Table 2**). Cholesteryl octonate-1- ^13^C_1_ is a cholesteryl ester not described in humans and was used as an internal standard (IS).

**Table 2:** Cholesteryl Esters Described in this Study.

Common name		Fatty acid residue ^a^	Mass
Cholesteryl -	octonate ^b^	C8:0	531.5
	palmitate	C16:0	642.6
	palmitoleate	C16:1	640.5
	stearate	C18:0	670.5
	oleate	C18:1	668.6
	linoleate	C18:2	666.6
	linolenate	C18:3	664.5
	arachidate	C20:0	698.5
	paullinate	C20:1	696.5
	arachidonate	C20:4	690.6

^a^ Carbon number: number of double bonds;

^b^ in stable ^13^C1 isotope form.

### Lipid Extraction

BALF samples (100 μL) were diluted with dH_2_O (1.7 mL) in glass screw-capped test tubes, the IS (250 pmol in 25 μL of dichloromethane) was added, and total lipid extracts were prepared as described previously [13,28]. Extracts were dried in a water bath at 35°C under a gentle stream of N_2_ gas, and stored at -20°C in the dark until further analysis by liquid chromatography/tandem mass spectrometry with multiple reaction monitoring.

### Liquid Chromatography/Tandem Mass Spectrometry (LC/MS/MS)

Liquid chromatography was used with tandem MS to identify and quantify individual cholesteryl esters. Dried lipid extracts from 100 μL BALF were re-dissolved in MeOH/CHCl_3_ containing 5 mM ammonium acetate (50/50, v/v, 50 μl). Aliquots (typically 5 μl) were injected onto a reverse phase HPLC column (Phenomenex Kinetex XB-C18, 100 x 2.1 mm, 1.7 μm particle size, 100 Å pore diameter) equilibrated in solvent A (MeOH/H_2_O, 90/10, v/v, containing 5 mM ammonium acetate) and eluted with an increasing concentration of solvent B (CHCl_3_/H_2_O, 500/0.2, v/v, containing 5 mM ammonium acetate: min/%B/μl per min; 0/0/200, 3/0/200, 3.01/0/100, 20.5/100/100, 23/100/100, 24/0/200, 30/0/200). The effluent from the column was directed to an electrospray ion source (Agilent Jet Steam, Agilent, Santa Clara, CA) coupled to a triple quadrupole mass spectrometer (Agilent 6460) operating in the positive ion multiple reaction monitoring (MRM) mode in which the intensity of pre-selected (M+NH_4_)^+^ parent→fragment ion transitions (cholesteryl-C20:0, 698.5→369.4;-C20:1, 696.5→369.4;-C20:4, 690.6→369.4;-C18:0, 670.5→369.4;-C18;1, 668.6→369.4;-C18:2, 666.6→369.4,-C18:3, 664.5→369.4;-C16:0, 642.6→369.4;-C16:1, 640.5→369.4; and ^13^C_1_-cholesteryl-C8:0 internal standard (IS), 531.5→369.4; **Table 2**) were recorded using instrument manufacturer-supplied software (Agilent MassHunter). With each batch of samples a series of standards were prepared containing the same amount of IS (250 pmol) and varying amounts of authentic cholersteryl-C20:4,-C18:0,-C18:1,-C18:2, and C16:0 (0, 5, 10, 50, and 100 pmol, all in duplicate). The amount of each CE in each sample was calculated by interpolation from the curves constructed using the data for the standard samples (ordinate, ratio peak area cholesteryl ester/peak area of the IS; abscissa, pmol of each authentic ester). Those species for which no authentic standard was available, the curve for the species with the closest mass was used. The detection limit of this method is 20–50 fmol per injection.

### Total Lipid Quantification with Nile Red

The fluoroprobe Nile red [29] (Sigma-Aldrich) was used to quantify total lipids in BALF. Ten μL of a Nile red stock solution (100 μg/mL in DMSO) was added to 90 μL BALF in duplicate in a 96-well flat bottom black microtiter plate (Corning Life Sciences—Axygen Inc., Union City, CA). After incubation in the dark for 10 min the relative fluorescence was quantified using a TECAN fluorescence reader (excitation at 485 nm, emission at 535 nm; Genios 2760063, TECAN Systems, San Jose, CA).

### Total Protein Quantification

To determine total protein concentration in BALF, the Bicinchoninic acid (BCA) Protein Assay Kit (Thermo scientific, Rockford, IL) was used according to the manufacturer's instructions following the microtiter plate procedure with bovine serum albumin as the standard.

### Quantification of Albumin

A sandwich ELISA colorimetric assay (Bethyl Laboratories, Inc., Montgomery, TX) was employed to quantify albumin in BALF according to the manufacturer's instructions using a 96-well flat bottom microtiter plate (Nunc Nalgene International, Rochester, NY).

### Western Immunoblot Quantification of HNP1-3

To quantify the antimicrobial peptide HNP1-3, BALF was subjected to SDS-PAGE followed by Western Immunoblotting as described previously [14,30] using a polyclonal rabbit anti-HNP1-3 antibody (1:400 dilution, kindly provided by Dr. Tomas Ganz, UCLA). The concentration of HNP1-3 was interpolated from a standard curve derived from purified HNP2 peptide (also provided by Dr. Tomas Ganz) using the Versadoc Imaging System and QuantityOne Software (BioRad, Hercules, CA). The polyclonal antibody is cross-reactive with all three major HNP forms.

### Data Analysis

Raw data were initially analyzed with Excel 2013 and Sigmaplot version 9.0 was used for graphing. IBM SPSS version 20.0 was used for statistical analysis and unless stated otherwise. Statistical significance was determined by first testing for equality of variances using Levene’s test. If there was no evidence of unequal variances, a one-tailed Student’s t-test for independent samples was used; otherwise, Welch's t-test for unequal variances was used. Probability levels less than 0.05 were flagged as statistically significant. With the exception of calculating statistical significance for differences in age, BALF volume, and infection status, statistical tests were run after log transformation of the data (log_10_ [1+data]).

## Results

### Study population and BALF characteristics

**Table 1** shows disease status, genotype for CF, age, and BALF characteristics for the 6 cystic fibrosis (CF) and 7 non-CF disease control pediatric subjects (Non-CF). There was a statistically significant difference between Non-CF and CF subjects in age (5.8 ± 1.6 *versus* 13.3 ± 1.4 years, means ± S.E.M, *p* = 0.007) and BALF volume (7.93 ± 0.41 versus 19.5 ± 4 mL, means ± S.E.M, *p* = 0.035), respectively). There was no statistically significant association between disease (Non-CF *versus* CF) and infection status (*p* = 0.500 using Fisher’s one-sided exact test for a 2x2 contingency table). Compared to Non-CF, cell counts were elevated in BALF collected from CF subjects, namely nucleated cells 288 ± 78 *versus* 1732 ± 721, red blood cells 178 ± 117 *versus* 1331 ± 777, and neutrophils 32 ± 12 *versus* 1253 ± 618 (cells/ μL for Non-CF *versus* CF, means ± S.E.M, respectively), and for nucleated cells and neutrophils the differences reached statistical significance after log transformation (*p* = 0.021 and *p* = 0.003, respectively).

### CE identification and quantification

**Fig 1:**
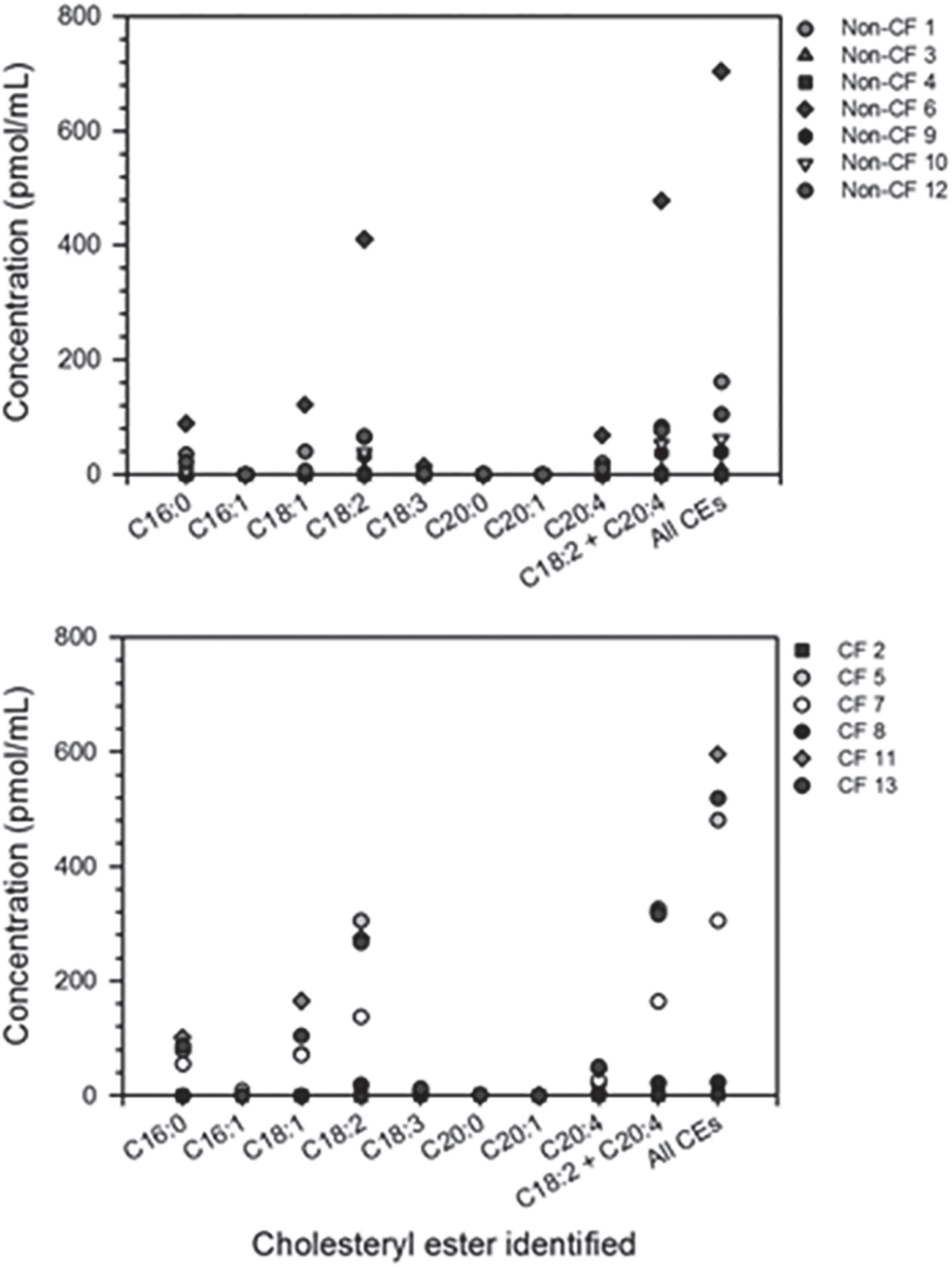
CE concentrations in BALF collected from non-CF disease control subjects (top) and CF patients (bottom). CEs are identified by the fatty acid species (number of carbons: number of unsaturated bonds) attached to the cholesterol molecule (C). Individual CE concentrations are given for each molecular species identified as well as for cholesteryl linoleate (C18:2) and cholesteryl arachidonate (C20:4) combined and all CEs combined.

On the basis of mass concordance and HPLC retention time, the molecular species of CEs detected in BALF were palmitate (16:0), palmitoleate (16:1), oleate (18:1), linoleate (18:2), linolenate (18:3), arachidate (20:0), paullinate (20:1) and arachidonate (20:4) (**Table 2**). The CE profile for each patient is shown in **Fig 1**. The most abundant CE was linoleate, which averaged 88.5 ± 54.5 pmol/mL in Non-CF BALF and 166.9 ± 55.3 pmol/mL in CF BALF (means ± S.E.M.). The next most abundant esters were oleate (24.0 ± 17.1 pmol/mL in Non-CF, and 68.8 ± 25.8 pmol/mL in CF) and palmitate (21.5 ± 12.4 pmol/mL in Non-CF disease controls, and 53.2 ±17.9 pmol/mL in CF). Cholesteryl arachidonate concentrations were 16.7 ± 9 pmol/mL in Non-CF and 24.5 ± 8.4 pmol/mL in CF. Cholesteryl linolenate levels averaged 2.8 ± 2 pmol/mL in Non-CF and 5.0 ±1.8 pmol/mL in CF. Of these, the linoleate and arachidonate esters are known to have antimicrobial activity against *P*. *aeruginosa* [13]. Signals corresponding to the palmitoleate, arachidate, and paullinate esters were detected, but at very low levels (< 2 pmol/mL). None of the observed differences in raw cholesteryl ester concentrations reached statistical significance and we queried their levels relative to total lipid.

### CEs adjusted to lipid content

**Fig 2:**
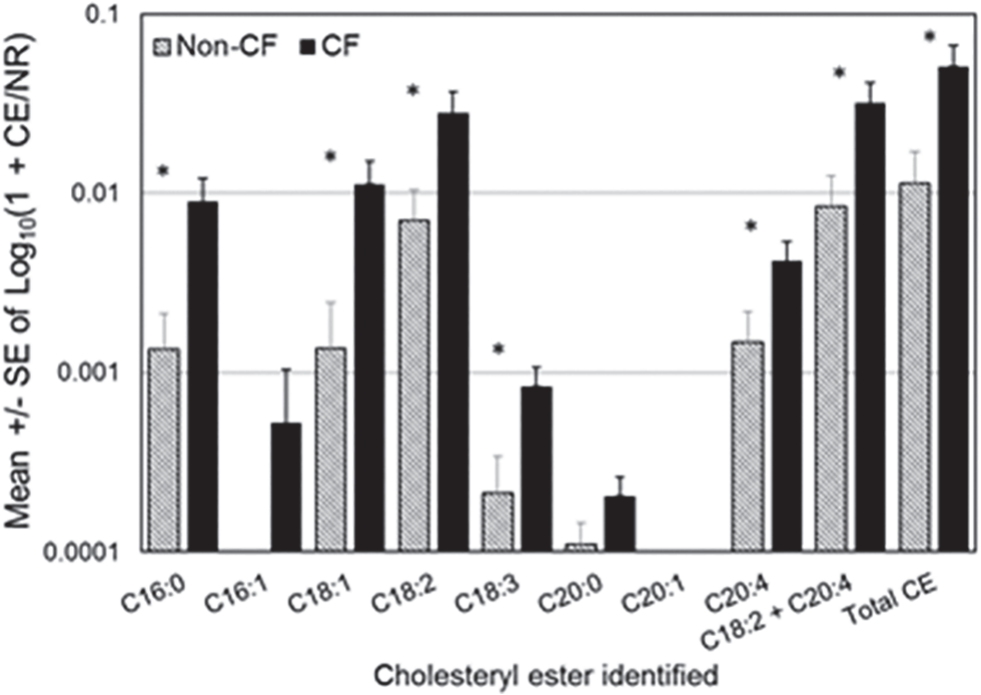
CE fraction of total lipid. CEs are identified by the fatty acid species (number of carbons: number of unsaturated bonds) attached to the cholesterol molecule (C). Individual CE concentrations are given for each molecular species identified as well as for cholesteryl linoleate (C18:2) and cholesteryl arachidonate (C20:4) combined and all CEs combined. Shown are the means ± S.E.M of log transformed data with n = 7 for Non-CF and n = 6 for CF. * indicates a *p* value of < 0.050 in one-tailed *t*-test.

To quantify total lipid content, a protocol that employs the fluoroprobe Nile red was adapted [29]. Nile red emits red fluorescence in lipophilic environments, thus as more lipid is present red fluorescence increases. Total lipid content did not significantly differ between CF and Non-CF (**Table 3**), but the ratio of total lipid to total protein was significantly decreased in CF BALF (25.9 ± 10.1 *versus* 6.7 ± 1.2, means ± S.E.M. of Non-CF *versus* CF, *p* = 0.018) and when CE concentrations were adjusted to total lipid, the CEs of palmitate (*p* = 0.03), oleate (*p* = 0.028), linoleate (*p* = 0.021), linolenate (*p* = 0.016), and arachidonate (*p* = 0.038), as well as cholesteryl linoleate and cholesteryl arachidonate combined, and all CEs combined, were significantly elevated in CF compared to Non-CF (**Table 4 and Fig 2**). We then questioned whether this increase correlates with inflammation.

**Table 3:** Total Lipid and Protein Analysis of BALF.

Disease	Subject Identifier	Total Lipid (Nile Red RFU ^a^)	Total Protein (μg/mL)	Albumin (μg/mL)	HNP1-3 (μg/mL)
Non-CF	1	18839.5	222.70	16.81	0.37
	3	1968.5	85.60	12.25	ND ^b^
	4	1245.5	71.97	9.72	ND
	6	6638.5	282.03	69.88	1.45
	9	2811.0	457.61	200.71	ND
	10	1585.0	208.27	19.62	1.78
	12	6185.5	330.14	35.75	ND
CF	2	1502.5	152.95	9.44	2.41
	5	2086.5	548.21	80.16	43.48
	7	1301.5	334.14	35.52	5.19
	8	935.0	205.06	35.68	1.12
	11	3724.5	367.02	16.24	37.29
	13	4472.5	577.88	32.92	34.88

^a^ RFU: relative fluorescence units;

^b^ ND: not detectable.

**Table 4:** Significance Levels for Total Lipid Adjusted Cholesteryl Ester Concentrations in BALF from Non-CF vs. CF Pediatric Patients.

CE ^a^		Levene's Test for Equality of Variances (Significance)	*t*-test for Equality of Means (Significance one-tailed)
C16:0	Equal variances assumed	.008	.014
	Equal variances not assumed		**.030**
C16:1	Equal variances assumed	.020	.150
	Equal variances not assumed		.182
C18:1	Equal variances assumed	.009	.013
	Equal variances not assumed		**.028**
C18:2	Equal variances assumed	.070	**.021**
	Equal variances not assumed		.035
C18:3	Equal variances assumed	.193	**.016**
	Equal variances not assumed		.024
C20:0	Equal variances assumed	.136	.092
	Equal variances not assumed		.103
C20:1	Equal variances assumed	.345	.292
	Equal variances not assumed		.284
C20:4	Equal variances assumed	.435	**.038**
	Equal variances not assumed		.048
CA + CL	Equal variances assumed	.082	**.021**
	Equal variances not assumed		.033
Total CE	Equal variances assumed	.027	.015
	Equal variances not assumed		**.028**

^a^ For each cholesteryl ester the fatty acid residue esterified to cholesterol is given. One-tailed significance was calculated with log_10_ (1+ [CE concentration as pmol per 1 RFU Nile red]) testing whether the individual CEs are elevated in CF compared to Non-CF. CL: cholesteryl linoleate; CA: cholesteryl arachidonate. P-values indicating statistical significance are shown in bold.

### Markers of inflammation

Inflammation is typically accompanied by an increase in total protein and increased plasma transudation to deliver additional host defense proteins, and an influx of immune cells, particularly neutrophils in CF. Albumin can be used to assess plasma transudation, and the antimicrobial peptides HNP1-3, major constituents of neutrophil granules, are reliable markers for neutrophil infiltration. In addition to neutrophil counts, these markers were used to assess the lung inflammation status of the subjects (**Table 3**), and then their relationship to CEs was determined.

Total protein and albumin concentrations did not differ significantly between Non-CF and CF subjects; total protein concentrations were 236.9 ± 51.3 *versus* 364.2 ± 70.8 μg/mL (*p* = 0.177) and albumin concentrations were 52.1 ± 26.0 *versus* 35.0 ± 10.1 μg/mL (*p* = 0.669), Non-CF *versus* CF, respectively. When CE concentrations were adjusted to total protein and albumin concentrations, there was no statistically significant difference between CF and Non-CF subjects.

**Fig 3:**
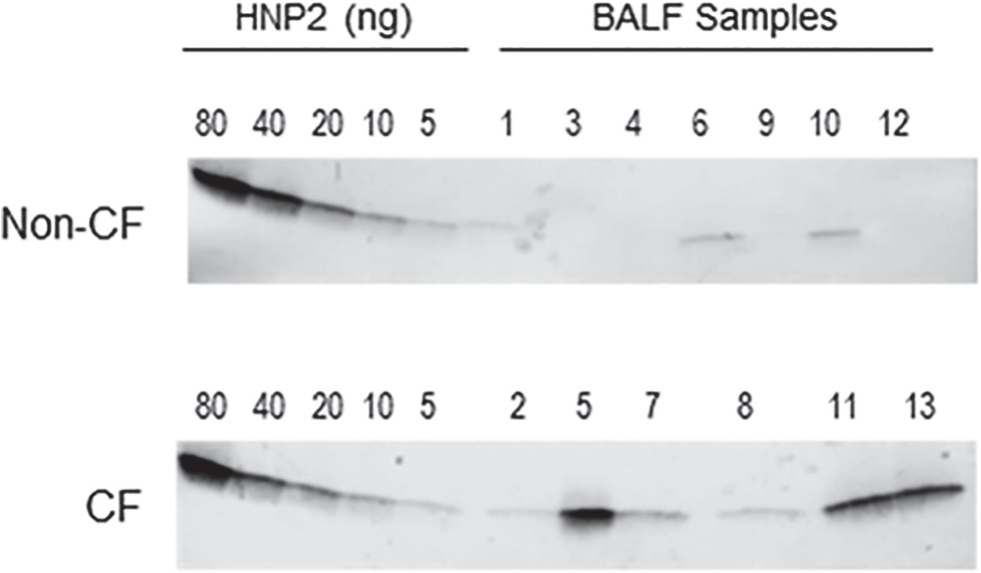
Quantification of HNP1-3 by Western immunoblot. HNP2 peptide standard and bronchoalveolar lavage fluid (BALF) samples were separated by 4–20% SDS PAGE, blotted onto PVDF-PSQ membranes, probed with polyclonal rabbit antibodies against HNP1-3, and antibody binding was visualized with goat-anti rabbit antibodies conjugated to alkaline phosphatase and NBT/BCIP substrate. HNP1-3 differ by one amino acid only and co-migrate in this gel system. Per lane, the equivalent of the following BALF volumes were loaded: for Non-CF samples, 1: 20 μL, 3: 40 μL, 4: 40 μL, 6: 20 μL, 9: 40 μL, 10: 20 μL, 12: 20 μL; for CF samples, 2: 6 μL, 5: 6 μL, 7: 6 μL, 8: 20 μL, 11: 6 μL, 13: 6 μL.

Next, HNP1-3 was quantified (**Fig 3 and S1 Fig. Original blots presented in cropped and gray tone version in Fig 3** and **Table 3**). HNP1-3 was present at low levels in Non-CF and significantly elevated in CF patients (0.52 ± 0.3 μg/mL *versus* 20.7 ± 8.1 μg/mL in Non-CF *versus* CF patients, means ± S.E.M., *p* = 0.01 before and *p* = 0.04 after adjustment to total protein). Furthermore, HNP1-3 concentrations as well as neutrophil counts correlated with the same CEs that represented an increased fraction of total lipid in CF patients (**Table 5**). In contrast, lung infection status did not correlate with any of the CEs (*p* values between 0.123 and 0.506).

**Table 5:** Correlation between Cholesteryl Esters and Markers of Inflammation.

	HNP		Neutrophil Count	
CE ^a^	Pearson Correlation	Significance (2-tailed)	Pearson Correlation	Significance (2-tailed)
C16:0	**0.661** *	**0.014**	**0.676** *	**0.011**
C16:1	0.105	0.733	0.212	0.487
C18:1	**0.699** **	**0.008**	**0.688** **	**0.009**
C18:2 (CL)	**0.575** *	**0.040**	**0.731** **	**0.005**
C18:3	**0.650** *	**0.016**	**0.758** **	**0.003**
C20:0	0.043	0.889	0.134	0.663
C20:1	0.005	0.988	0.009	0.976
C20:4 (CA)	**0.608** *	**0.028**	**0.775** **	**0.002**
CL + CA	**0.591** *	**0.033**	**0.763** **	**0.002**
Total CE	**0.614** *	**0.025**	**0.768** **	**0.002**

^a^ For each cholesteryl ester the fatty acid residue esterified to cholesterol is given. Correlation was calculated with log transformed data (log_10_ [1+data]).

*Correlation is significant at the 0.05 level (2-tailed).

**Correlation is significant at the 0.01 level (2-tailed). P-values indicating statistical significance are shown in bold. CL: cholesteryl linoleate; CA: cholesteryl arachidonate.

## Discussion

Cholesteryl linoleate and arachidonate have been recently identified as antimicrobial effectors in airway fluid, and cholesteryl linoleate concentrations have been found to be elevated in the context of inflammation [13,14]. This study suggests that in BALF collected from pediatric CF subjects, total lipid relative to total protein is decreased, and that when corrected for total lipid content palmitate, oleate, linoleate, linolenate, and arachidonate CEs are significantly elevated, compared to non-CF disease control subjects. Furthermore, CE concentrations correlated with HNP1-3 and neutrophil counts, both measures of inflammation. These data provide additional evidence that in CF airways the lipid composition is altered and that CEs take part in the inflammatory lung response inviting future studies to explore the potential of CEs as a novel biomarker for CF disease activity.

The CE species identified in BALF represented species previously identified in nasal fluid [13], with the exception of C20:1 which has yet not been described in human body fluids. However, the detected concentrations in BALF are low and considering the high sensitivity of the methodology used in this study, this CE species might have been present but undetected in earlier studies.

The total lipid to protein ratio was decreased in CF BALF. This is in line with earlier studies by Meyer *et al*., who described reduced phospholipid to protein ratios in lipid extracts of BALF collected from young adults with CF [31]. However, CEs of palmitate, oleate, linoleate, linolenate, and arachidonate were elevated in the lipid fraction of BALF collected from CF subjects. While it cannot be ruled out that the age differences in the study populations may have contributed to the observed differences, increased levels of CEs have also been found earlier in expectorated tracheobronchial secretions of CF patients [32], and it has been demonstrated that chronically infected CF patients have significantly higher CE levels in their plasma compared to non-chronically infected CF patients [33]. Decreased cholesteryl ester content of plasma lipoproteins in CF have been reported elsewhere [34,35] but in these studies CE content was indirectly calculated as the difference between total and unesterified cholesterol, and individual cholesteryl esters have not been quantified precluding detection of relative changes among the various CEs. Furthermore, the CE deficiency was attributed to defective hepatic lipase and a dysfunctional cholesteryl ester transfer protein representing metabolic changes of the liver while BALF used in this study reflects CE alterations in the airways.

We have shown previously that epithelial cells secrete CEs in the airways [13]. Here, CEs correlated with HNP1-3 and neutrophil counts, which both are strong indicators of inflammation [36,37]. Within the limitations of the small sample size of this study, CEs did not correlate with the infection status. Thus, their elevated levels do not appear to be simply a surrogate of infection but could reflect an up-regulation of their production and secretion by epithelia in the context of inflammation in CF, independent from infection. A neutrophil elastase-initiated modulation of epithelial cell transcription has been recently reported by Fischer *et al*. who observed an increased expression of senescence markers in CF airway tissue sections, and an *in vitro* up-regulation of these markers in neutrophil elastase-treated epithelial cells [38]. Alternatively, neutrophils could deliver CEs to the site of inflammation, but information on CEs in neutrophils is limited. May *et al* reported that total esterified cholesterol in neutrophils decreased after LPS stimulation [39], which could be consistent with CE secretion. For monocytes and macrophages, significant amounts of stored CEs and their increase after stimulation have been reported [15–17]. Thus, the elevated CE concentrations in BALF collected from CF subjects may be contributed by both epithelial and inflammatory cells. While increased hemorrhagic lesions and plasma transudation have been reported in CF disease [40], lack of statistically significant differences in red blood cells and albumin in BALF make this source less likely, but future studies that measure in parallel plasma and BALF concentrations of albumin and CEs are needed to ascertain this notion. This would also resolve the apparent discrepancy between plasma [34,35] and BALF CE levels as reported here.

While this study suggests that in pediatric CF patients the CEs of palmitate, oleate, linoleate, linolenate, and arachidonate are elevated in the lipid fraction of BALF compared to Non-CF, future studies are needed to determine whether antimicrobial CEs are active in the microenvironment of the CF lung. For example, it has been shown that while the concentrations of the antimicrobial peptide LL-37 are elevated in CF lungs, this antimicrobial peptide is inactive in the CF lung microenvironment [41,42]. Similarly, it has been recently reported that the pH of CF airway secretions is reduced and that the antimicrobial activity of lysozyme and other antimicrobial polypeptides is reduced at the pH observed in the CF fluids [9].

The findings of this study suggest that the observed increase in CEs in the lipid fraction of CF BALF reflects inflammation. Considering that increased CE levels and increased expression of a key enzyme for CE biosynthesis have been also found in chronic rhinosinusitis [14,43], the observed inflammation-associated increase in CEs is not CF specific. Thus, CEs may be explored as a novel biomarker classifying mild versus severe cases and monitoring disease activity. Using CEs as biomarkers of disease activity may add a measure of the epithelial cell response. There have been only few biomarkers described that are known products of epithelial cells, namely the proinflammatory cytokines IL-1β, IL-6, and IL-8 in sputum samples and the chemokine IP-10 in nasal lavage fluid [22,44–46], and chloride secretion [24,47]. A larger, age-controlled longitudinal study that measures in BALF known biomarkers of CF such as neutrophil elastase [22,23] in parallel with CEs is needed to confirm the suitability of CE levels as novel biomarker of CF disease activity that may provide new information on the lipid mediated pathophysiology of the disease.

## Conclusions

The lipid composition of BALF is altered in CF with less total lipid relative to protein but increased CE concentrations in the lipid fraction, likely contributed by inflammation. Future longitudinal studies may reveal the suitability of CEs as a novel biomarker for CF disease activity which may provide new information on the lipid mediated pathophysiology of the disease.

## Supporting Information

S1 Fig Original blots presented in cropped and gray tone version in Fig 3. The primary antibody is a polyclonal antibody that shows unspecific reactivity with higher molecular weight components in BALF.
(TIF)
