# Supplementary figures and images for: Cholesteryl Esters Are Elevated in the Lipid Fraction of Bronchoalveolar Lavage Fluid Collected from Pediatric Cystic Fibrosis Patients
Source: PLoS One. 2015 Apr 28;10(4):e0125326. doi: 10.1371/journal.pone.0125326 (PMC4412572; doi:10.1371/journal.pone.0125326)

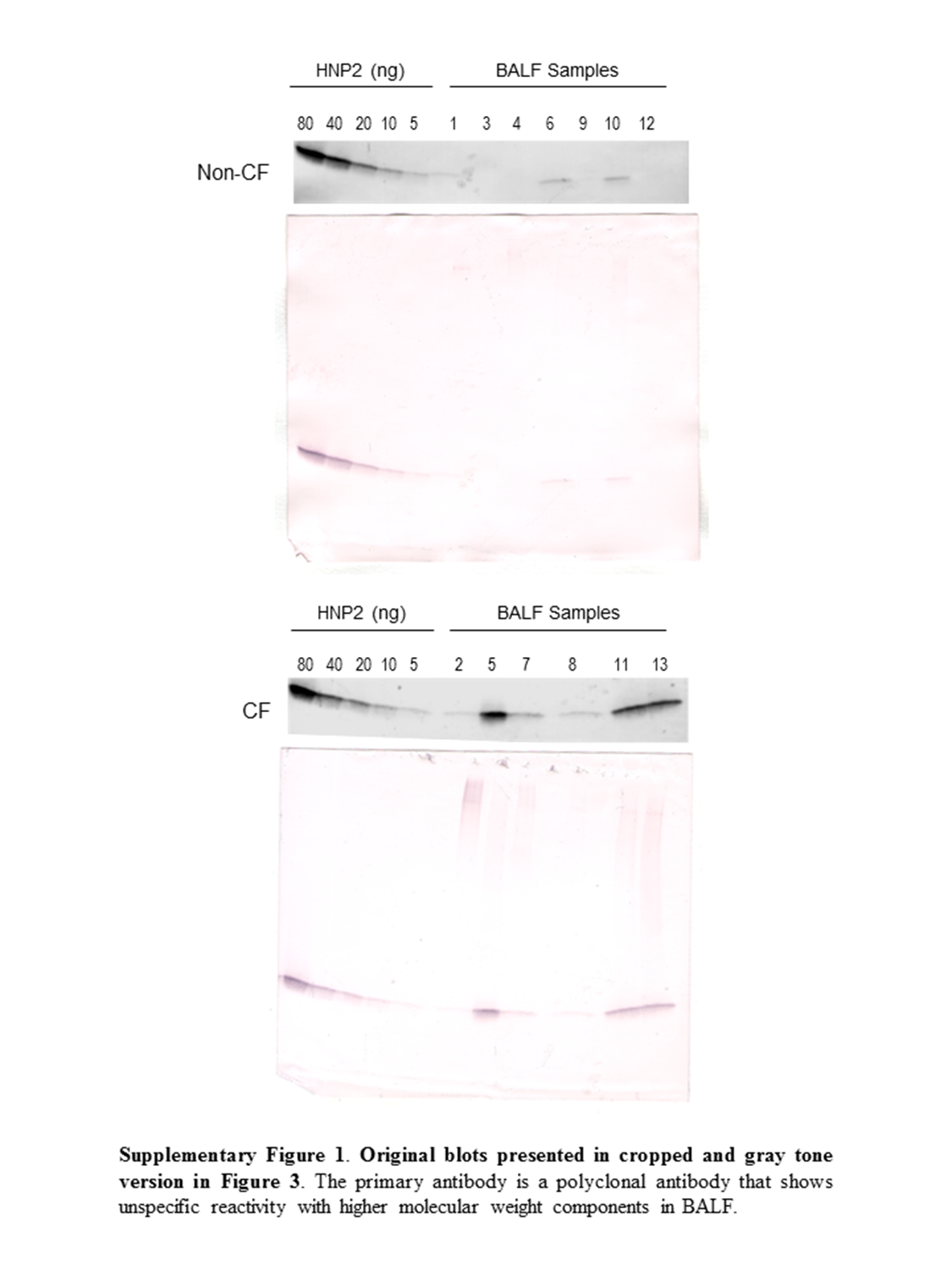

Supplement: S1 Fig — The primary antibody is a polyclonal antibody that shows unspecific reactivity with higher molecular weight components in BALF. (TIF) [file pone.0125326.s001.tif]
